# Treadmill Exercise before and during Pregnancy Improves Bone Deficits in Pregnant Growth Restricted Rats without the Exacerbated Effects of High Fat Diet

**DOI:** 10.3390/nu11061236

**Published:** 2019-05-30

**Authors:** Kristina Anevska, Dayana Mahizir, Jessica F. Briffa, Andrew J. Jefferies, John D. Wark, Brian L. Grills, Rhys D. Brady, Stuart J. McDonald, Mary E. Wlodek, Tania Romano

**Affiliations:** 1 Department of Physiology, Anatomy and Microbiology, LaTrobe University, Bundoora, VIC 3083, Australia; K.Anevska@latrobe.edu.au (K.A.); Brian.Grills@latrobe.edu.au (B.L.G.); Rhys.Brady@monash.edu (R.D.B.); Stuart.Mcdonald@latrobe.edu.au (S.J.M.); 2 Department of Physiology, The University of Melbourne, Parkville, VIC 3010, Australia; n.mahizir@student.unimelb.edu.au (D.M.); jessica.griffith@unimelb.edu.au (J.F.B.); Andrew.Jefferies@petermac.org (A.J.J.); m.wlodek@unimelb.edu.au (M.E.W.); 3 Department of Medicine, The University of Melbourne, Parkville, VIC 3010, Australia; jdwark@unimelb.edu.au; 4 Bone and Mineral Medicine, Royal Melbourne Hospital, Parkville, VIC 3050, Australia

**Keywords:** bone, high-fat feeding, growth restriction, exercise, pregnancy

## Abstract

Growth restriction programs adult bone deficits and increases the risk of obesity, which may be exacerbated during pregnancy. We aimed to determine if high-fat feeding could exacerbate the bone deficits in pregnant growth restricted dams, and whether treadmill exercise would attenuate these deficits. Uteroplacental insufficiency was induced on embryonic day 18 (E18) in Wistar Kyoto (WKY) rats using bilateral uterine vessel ligation (restricted) or sham (control) surgery. The F1 females consumed a standard or high-fat (HFD) diet from 5 weeks, commenced treadmill exercise at 16 weeks, and they were mated at 20 weeks. Femora and plasma from the pregnant dams were collected at post-mortem (E20) for peripheral quantitative computed tomography (pQCT), mechanical testing, histomorphometry, and plasma analysis. Sedentary restricted females had bone deficits compared to the controls, irrespective of diet, where such deficits were prevented with exercise. Osteocalcin increased in the sedentary restricted females compared to the control females. In the sedentary HFD females, osteocalcin was reduced and CTX-1 was increased, with increased peak force and bending stress compared to the chow females. Exercise that was initiated before and continued during pregnancy prevented bone deficits in the dams born growth restricted, whereas a HFD consumption had minimal bone effects. These findings further highlight the beneficial effects of exercise for individuals at risk of bone deficits.

## 1. Introduction

Pregnancy is associated with changes in the maternal skeleton to meet the needs of the developing fetal skeleton [1,2]. Dysregulation of these normal physiological adaptations to pregnancy occur in maternal undernutrition [3,4] and uteroplacental insufficiency [5,6,7,8,9,10]. Maternal undernutrition and uteroplacental insufficiency both lead to the development of a low birth weight baby, weighing less than 2.5 kg at term [11,12,13], with uteroplacental insufficiency affecting ~10% of pregnancies in the developed world [14]. Low birth weight individuals are at an increased risk of poor bone health (reduced bone mineral content (BMC) and density (BMD)), as well as obesity in adulthood, which is observed in both human [15,16] and animal models [8,9,17]. 

Our rat model of uteroplacental insufficiency, induced by bilateral uterine vessel ligation late in gestation to reduce fetal oxygen and nutrient transfer, mimics the 10–15% reduction in birth weight observed in humans [14,18,19], and it programs poor bone health in F0 dams and their F1 offspring. Specifically, rat dams (initial generation, F0) whose pregnancies were complicated by uteroplacental insufficiency did not lose bone during late gestation, which was required for mineralization of the fetal skeleton [7]. Consequently, the F1 male and female growth restricted offspring had reduced total body calcium on postnatal day 6 [20], and at 6 months of age, they have shorter femora with reduced trabecular and cortical content, as well as bending strength [6,8,9]. Interestingly, we have reported that F1 growth restricted females have normal skeletal adaptations during late gestation, with their F2 offspring having normal bone health [18]. This highlights that the programmed bone effects of growth restriction only influence the F1 generation. 

Studies in both humans and animals have reported clear links between a low birth weight and increased obesity risk [16,17]. Obesity has been reported to have beneficial effects on bone health in humans [21,22,23] and animals [24,25]. However, these findings are controversial with other studies reporting impaired bone health in obese humans [26,27,28], and in high-fat fed animal models [29,30,31]. On the other hand, some experimental models report no changes in bone health following high-fat feeding [32]. These discrepancies, especially in animal models, were likely due to differences in diet composition (fat and carbohydrate content), the timing and duration of dietary intervention, sex, and animal strain. Currently, the relationship between obesity and bone health in F1 growth restricted females, especially during pregnancy, remains to be elucidated. 

Although several studies use weight-bearing exercise, since it promotes bone formation [33,34], treadmill exercise is an effective intervention to improve bone outcomes [35,36]. Specifically, treadmill exercise in female rats increases the cortical BMD and bone mass in the periosteal envelope of the tibia [35], and it increases femoral length and tibial BMC [37]. More recently, moderate treadmill exercise was reported to have anabolic effects on bone in healthy male rats by increasing the trabecular bone mass and reducing trabecular separation; suggesting that exercise favors bone formation [38]. However, exercise effects on bone in F1 pregnant growth restricted females has not been investigated. Therefore, it is possible that exercise initiated prior to and maintained throughout pregnancy could be an effective intervention to offset the negative effects of growth restriction on bone in F1 growth restricted females. 

Therefore, this study aimed to determine whether high-fat feeding exacerbated the programmed bone deficits in growth restricted rat dams. We also aimed to characterize whether treadmill exercise initiated before and continued throughout pregnancy would prevent the bone deficits in growth restricted females during late pregnancy.

## 2. Materials and Methods 

### 2.1. Animals

The University of Melbourne Animal Ethics Committee, (AEC number 1212639) approved all the animal experimental procedures. Female F0 Wistar-Kyoto rats (initial generation, F0) were purchased from the Biological Research Facility (The University of Melbourne) at 8 weeks of age, and they were cohoused (6 rats/box) in a temperature-controlled room (22 °C), with a 12 h light-dark cycle and access to standard rat chow and tap water ad libitum. At 16 weeks of age, the F0 females were mated with breeder males. On gestation day 18 (E18), the dams were allocated to either uteroplacental insufficiency (bilateral uterine vessel ligation; offspring termed: restricted) or sham (offspring termed: control) surgery, as previously described in Reference [19]. The dams delivered pups (F1, first generation) at term on E22. At 5 weeks of age, the F1 control and restricted sibling females (maximum 2 siblings/litter/diet) were randomly allocated to a chow diet (AIN93G containing 7% fat) or HFD (SF01-028 containing 23% fat, and SF03-020 containing 23% fat and 0.19% cholesterol) obtained from Specialty Feeds, Australia. The females consumed these allocated diets for the remainder of the experiment. 

### 2.2. Exercise Protocol

**Figure 1:**
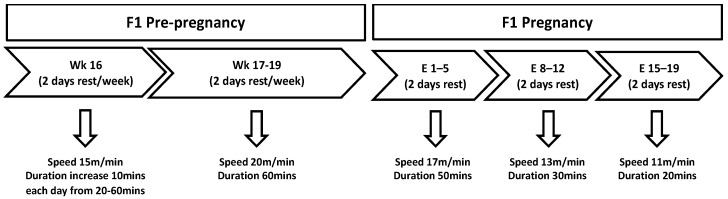
Exercise protocol during pre-pregnancy: F1 females began exercising at 16 weeks, where the intensity and duration increased each day. From 17 weeks, the females ran for the same duration and time, for 5 days a week with 2 days rest until mating. During pregnancy, the exercise intensity and duration decreased, but the females continued to run for 5 days per week followed by 2 days of rest.

At 16 weeks of age, the F1 control and restricted sibling females were randomly allocated to either remain sedentary or to commence treadmill exercises (exercise). This allocation gave rise to 8 experimental groups, each with 1 female sibling/group: control-chow-sedentary (n = 10), restricted-chow-sedentary (n = 12), control-HFD-sedentary (n = 10), restricted-HFD-sedentary (n = 10), control-chow-exercise (n = 8), restricted-chow-exercise (n = 9), control-HFD-exercise (n = 8), and restricted-HFD-exercise (n = 8). The treadmill exercise was performed as previously described in Reference [39]. Briefly, the females allocated to the exercise group ran for 5 days/week with 2 days of rest for 4 weeks on a motorized treadmill (Columbus Instruments, Columbus, OH, USA) at a 0° incline prior to pregnancy [39]. On day 1 of the exercise protocol, the F1 females ran for 20 min at a speed of 15 m/min, and on each subsequent day, the exercise duration was increased by 10 min such that on day 5 of the protocol, the rats were exercised for 60 min [39] (Figure 1). From week 2 of the protocol, the rats ran for 60 min at 20 m/min (Figure 1). Once pregnant, the exercise duration and intensity were decreased with each week of pregnancy [40]: In week 1, the rats ran for 50 min at 17 m/min; in week 2 rats, the rats ran for 30 min at 13 m/min; and in week 3, the rats ran for 20 min at 11 m/min (Figure 1).

### 2.3. Post-Mortem Blood and Tissue Collection 

On E20, all the F1 dams were euthanized with an intraperitoneal injection of Ketamine (100 mg/kg body weight) and Ilium Xylazil-20 (30 mg/kg body weight). Major organs, tissues, and muscles were excised and weighed. The left and right femora were dissected and measured using digital calipers, and then stored at 4 °C in silicon oil and 0.1 M sodium cacodylate buffer, respectively. Blood was collected via cardiac puncture, followed by treatment with heparin, and then centrifuged for 15 min at 3000 rpm. Plasma was collected for bone marker, adiponectin, and leptin assays, and then stored at −20 °C. 

### 2.4. Peripheral Quantitative Computed Tomography (pQCT) 

Bone scans were performed on the right femora collected at E20 from all the F1 dams using pQCT (Stratec XCT-Research SA+. Stratec Research Pty. Ltd., Pforzheim, Germany), along with the accompanying software, as previously described in References [18,41]. Briefly, each femur was individually placed into a plastic tube (7.5 cm × 1.2 cm) for accurate alignment within the specimen holder of the pQCT. Prior to scanning, a low-resolution scout scan was performed and a reference line was placed at the upper border of the distal condyle of the femur. A 1 mm slice (voxel size 0.1000 mm^3^, peel mode 20, contour mode 1) was taken at distances from the reference line of 5% to quantify trabecular bone, and at 50% to quantify cortical bone. Automatic density thresholding (400 mg/cm^3^) was used to eliminate the effect of any soft tissue remaining on the femur after dissection. Tissue density of 280 mg/cm^3^ or less was classified as trabecular bone, and tissue density of 710 mg/cm^3^ or greater was classified as cortical bone. 

### 2.5. Histological Processing, Staining, and Histomorphometry

Prior to histological processing, the scanned right femora of the F1 dams were fixed in 4% paraformaldehyde and 0.1 M sodium cacodylate fixative for 48 h, followed by 3 washes for 30 min, and then stored in a solution containing 0.1 M sodium cacodylate and 10% sucrose [42]. The femora were processed to plastic, then sectioned and stained. Briefly, the right femora were dehydrated using graded concentrations of ethanol (70%, 90%, and 100%), and the samples were infiltrated and embedded in LR White resin (London Resin Company limited, Reading, England) and polymerized in LR White resin at 60 °C for 24 h. Longitudinal plastic sections were cut at 5 µm, at the midpoint of the undecalcified femora using a tungsten carbide blade on a Leica RM 2155 Rotary Microtome (Leica, Wetzlar, Germany). Sections were stained with Light Green and then counter-stained with Safranin O to examine bone and cartilage content [42]. Stained sections were photographed at 25× magnification using Leica IM50 imaging software (Heerbrugg, Switzerland), and then viewed using a Leica DFC420 Light Microscope (Heerbrugg, Switzerland). The following parameters were measured: growth plate thickness (µm), trabecular bone area (percentage), and calcified cartilage (percentage), using the Leica Qwin V3 Standard software (Heerbrugg, Switzerland).

### 2.6. Mechanical Testing

Testing was performed as described previously in References [42,43]. Briefly, the left femora of the F1 dams were individually placed onto the three-point bending apparatus in a mediolateral position. The stabilizing platform consisted of a 16 mm gap. The fulcrum was kept at a constant rate of 100 mm/min, and peak stress was applied to the center of the femur in a mediolateral direction. The force measured (g) and deflection (mm) were recorded from the force transducer. These values were converted to Newton’s (N), and the displacement to meters (m).

### 2.7. Plasma Analysis

Rat-specific standard enzyme-linked immune-sorbent assays (ELISAs) were used to determine the plasma concentrations of osteocalcin (both uncarboxylated and carboxylated forms) (Rat-MID Osteocalcin ELISA, Immuno-Diagnostics Systems), C-terminal telopeptides of type 1 collagen (CTX-1 RatLaps ELISA, Immuno-Diagnostics Systems), leptin (Signosis Inc., Santa Carla, CA, USA), and adiponectin (R&D Systems, Minneapolis, MN, USA) in the F1 dams at E20. The intra-assay and inter-assay coefficients of osteocalcin were 4% and 6.7%, respectively, with a detection limit of 50.0 ng/mL. The intra-assay and inter-assay coefficients of CTX-1 were 6.8% and 12%, respectively, with a detection limit of 2.0 ng/mL. Plasma leptin intra-assay and inter-assay coefficients were 1.4–4.6% and 8.5–9.4%, respectively. Adiponectin intra-assay and inter-assay coefficients were 0.4–1.6% and 6.5–7.8%, respectively. 

### 2.8. Statistical Analyses

Data are presented as mean ± standard error of mean (SEM), with n denoting the number of animals per group. A normality test was first performed on all the data sets to identify if they were normally distributed. Maternal F1 data were first analyzed using a two-way ANOVA (SPSS-X; SPSS, Armonk, NY, USA) to determine the main treatment (control and restricted) and diet (chow and HFD) effects within each exercise regime. If interactions were observed, the data was split to identify the treatment effects within each diet, and the diet effects within each treatment, using the Student’s unpaired *t*-tests. The data was then split by treatment and an additional two-way ANOVA was performed to report the main exercise (sedentary and exercise) and diet (chow and HFD) effects within each treatment. If an interaction was observed, the data was split to identify the exercise effects within each diet and the diet effects within each exercise using the Student’s unpaired *t*-tests. Statistical significance was set at *p* < 0.05. 

## 3. Results

### 3.1. Body Weight

Unsurprisingly, the F1 restricted females were lighter at birth than the control females (*p* = 0.0001, −16%, Table 1). Restricted females remained lighter than the controls at postnatal days 7 (PN7), 14, and 35 (*p* = 0.0001, −10–19%, Table 1). Regardless of diet and exercise, the restricted females remained lighter at 15 and 19 weeks of age, at the time of mating (20 weeks), and at post-mortem (Table 2). Sedentary restricted females also gained less weight during pregnancy than the controls (Table 2), irrespective of diet. Sedentary females consuming a HFD, regardless of birth weight, were heavier than the chow-fed females at 19 weeks (Table 2) and at post-mortem (Table 2). Meanwhile, in the exercise cohort, the HFD fed females were heavier at 15 weeks, 19 weeks, mating, throughout pregnancy (Table 2), and post-mortem (*p* < 0.05, Table 2). 

### 3.2. Peripheral Quantitative Computed Tomography

**Figure 2:**
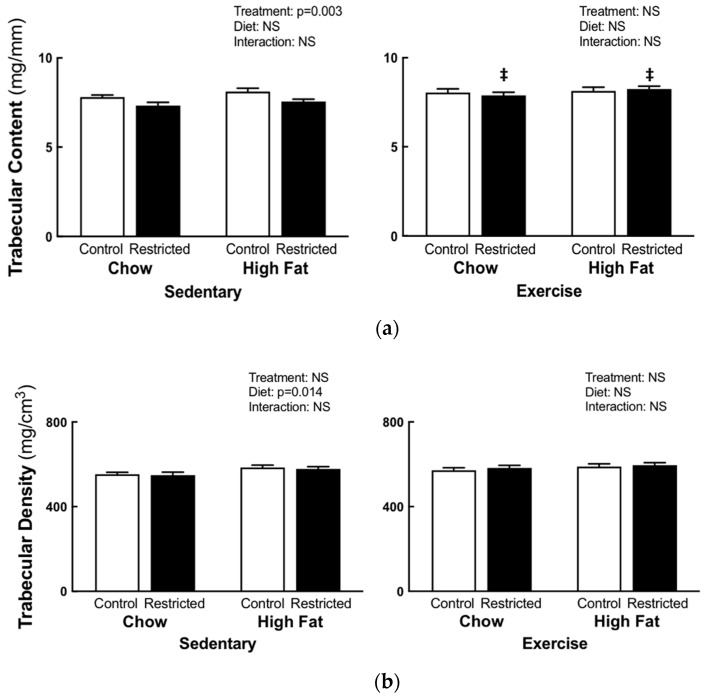
F1 maternal pQCT trabecular content and density. pQCT trabecular content (**a**) and density (**b**) at E20. Statistical significance (*p* < 0.05) determined by two-way ANOVA to identify the differences between treatments (control and restricted) and diets (Chow and HFD) within each exercise regime. A separate two-way ANOVA was then performed to identify the exercise and diet effects within each treatment (control and restricted). ‡ *p* < 0.05 vs. restricted sedentary. NS represents no statistical significance. Data is presented as mean ± SEM (n = 8–12/group).

**Figure 3:**
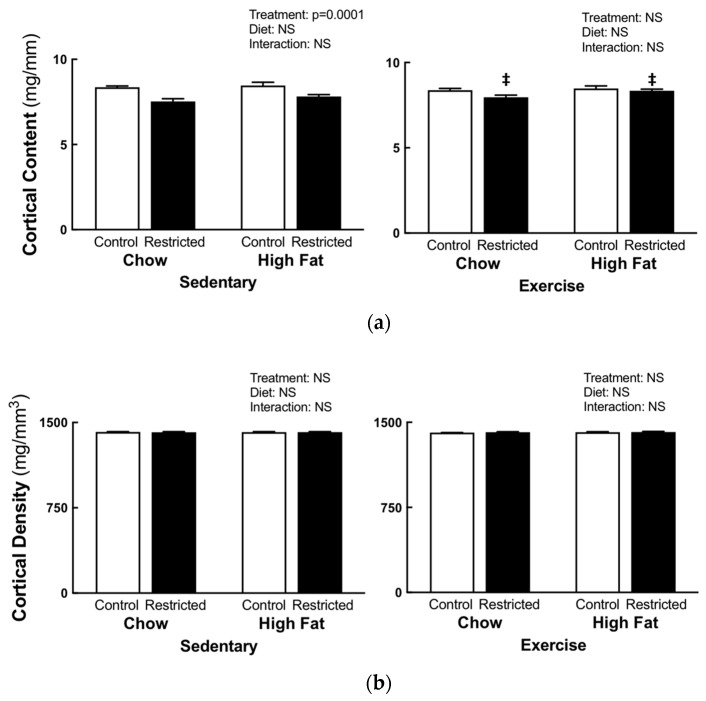
F1 maternal pQCT cortical content and density. pQCT cortical content (**a**) and density (**b**) at E20. Statistical significance (*p* < 0.05) determined by two-way ANOVA to identify the differences between treatments (control and restricted) and diets (chow and HFD) within each exercise regime. A separate two-way ANOVA was then performed to identify the exercise and diet effects within each treatment (control and restricted). ‡ *p* < 0.05 vs. restricted sedentary. NS represents no statistical significance. Data is presented as mean ± SEM (n = 8–12/group).

**Figure 4:**
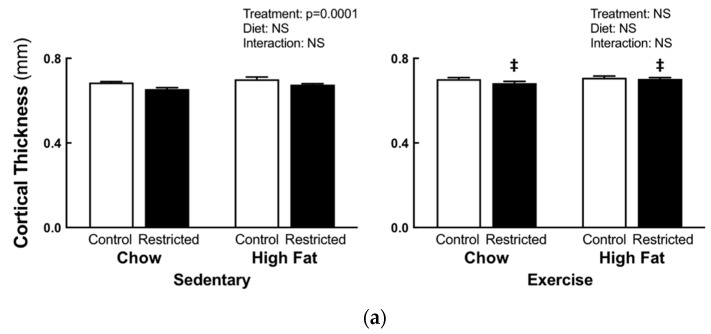

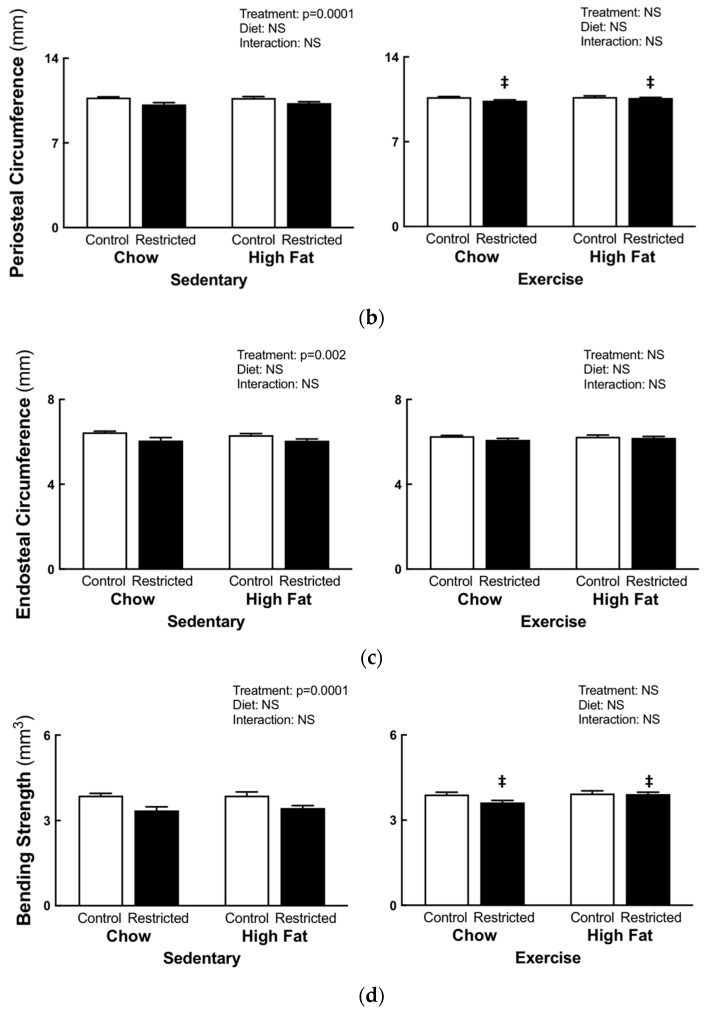
F1 maternal pQCT bone geometry measures. pQCT bone geometry measures cortical thickness (**a**), periosteal circumference (**b**), endosteal circumference (**c**), and bending strength (**d**) at E20. Statistical significance (*p* < 0.05) determined by two-way ANOVA to identify the differences between treatments (control and restricted) and diets (chow and HFD) within each exercise regime. A separate two-way ANOVA was then preformed to identify the exercise and diet effects within each treatment (control and restricted). ‡ *p* < 0.05 vs. restricted sedentary. NS represents no statistical significance. Data is presented as mean ± SEM (n = 8–12/group).

Sedentary restricted females, compared to the control, had decreased trabecular (−6.4%, Figure 2a) and cortical (−9%, Figure 3a) content, cortical thickness (−4%, Figure 4a), periosteal (−5%, Figure 4b), and endosteal (−5%, Figure 4c) circumference, and bending strength (−13%, Figure 4d), irrespective of diet. Sedentary HFD females had increased trabecular density compared to the sedentary chow females (+5.5%, Figure 2b); with no differences in cortical density (Figure 3b). Two-way ANOVA comparisons of the pQCT measures were not significantly different in the exercise groups. When comparing the restricted sedentary and exercise females, treadmill exercise increased the trabecular (*p* = 0.001, +8.4%, Figure 2a) and cortical (*p* = 0.0001, +6.3%, Figure 3a) content, cortical thickness (*p* = 0.0001, +4.2%, Figure 4b), periosteal circumference (*p* = 0.028, +2.5% Figure 4b) and, importantly, the bending strength (*p* = 0.001, +11.11%, Figure 4d). 

### 3.3. Histomorphometric Analysis and Mechanical Testing 

Data obtained from the histomorphometric analysis did not display differences in bone area, calcified cartilage, or growth plate size (Table 3). The three-point bending test yielded an increase in the peak force (*p* = 0.003, +15%) and bending stress (*p* = 0.003, +17%) in the HFD Sedentary females (Table 4), compared to the chow sedentary females, irrespective of their birth weight. There were no differences in the exercise cohort. 

### 3.4. Plasma Analysis

**Figure 5:**
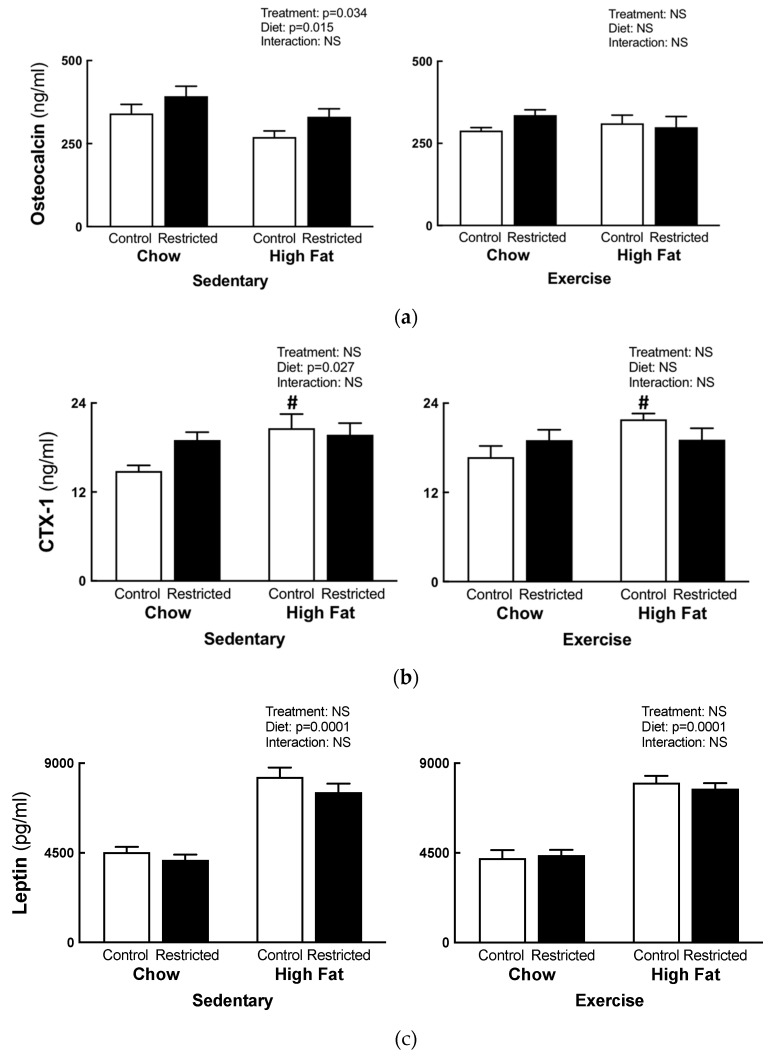
F1 maternal bone turnover markers. Plasma markers of bone turnover Osteocalcin (**a**), CTX-1 (**b**), and Leptin (**c**) at E20. Statistical significance (*p* < 0.05) determined by two-way ANOVA to identify the differences between treatments (control and restricted) and diets (chow and HFD) within each exercise regime. A separate two–way ANOVA was then preformed to identify the exercise and diet effects within each treatment (control and restricted). # *p* < 0.05 vs. chow. NS represents no statistical significance. Data is presented as mean ± SEM (n = 8/group).

Osteocalcin concentrations were increased in the sedentary restricted females (+18%, Figure 5a), irrespective of their diet. Sedentary HFD females had decreased osteocalcin concentrations (−18%, Figure 5a), irrespective of the maternal birth weight, with no differences observed in the exercise cohort. CTX-1 concentrations were increased in the sedentary HFD females compared to the chow females, irrespective of birth weight (+19%, Figure 5b), but they were not different in the exercise cohort. When the data was split by treatment, the control HFD females showed increased CTX-1 concentrations (*p* = 0.0001, +19%, Figure 5b) compared to the chow-fed controls. There were no differences in the plasma adiponectin concentrations (data not shown). The plasma leptin concentrations were increased in the sedentary HFD (*p* = 0.0001, +83%) and exercise HFD females (*p* = 0.0001, +83%) compared to their chow-fed counterparts (Figure 5c).

## 4. Discussion

To our knowledge, this was the first study to report that treadmill exercise performed before and continued throughout pregnancy increases trabecular and cortical content, cortical thickness, periosteal circumference, and bending strength in F1 growth restricted females. However, it is unclear whether these beneficial effects of exercise on the maternal skeleton can be sustained once exercise ceases or post-pregnancy. Furthermore, a HFD consumption did not cause an overt obesity phenotype in our model nor did it exacerbate the reported bone deficits in restricted females. 

### 4.1. Bone Health in Pregnant Growth Restricted Females

Under normal circumstances, the ratio of bone formation and resorption is coupled to maintain bone integrity [44]. Hormones, such as leptin acting on the CNS [45] and osteocalcin, as well as other mediators (CTX-1), influence the maintenance of normal bone mass [44]. In the present study, osteocalcin and CTX-1 were measured to quantify bone formation and resorption, respectively. Despite bone deficits, as measured by pQCT, the sedentary restricted females had increased plasma osteocalcin concentrations. Prior studies have reported that during late gestation, on day 21 of a 22 day pregnancy, healthy Wistar rats had increased plasma osteocalcin concentrations [46], suggesting that this normal physiological adaptation was exaggerated in restricted dams. The exact mechanism for this increase is unknown; however, it may be driven by the developing fetuses’ need for skeletal mineralization or to maintain fetal development, since maternal osteocalcin can cross the placenta and influence fetal brain development [47]. Furthermore, osteocalcin is believed to be under the hormonal control of insulin and leptin [48]. Since leptin concentrations were not different between the control and restricted dams, it is unlikely to have been responsible for the increased osteocalcin concentrations observed in the current study. However, we cannot discount the alterations in leptin resistance on bone health [45,49] or the effect of insulin on osteocalcin concentrations, which requires further investigation. 

### 4.2. Bone Health Following High-fat Feeding

With regards to high-fat feeding, the osteocalcin concentrations were reduced (sedentary only) and CTX-1 concentrations were increased (sedentary only and control dams). This finding was consistent with previous studies which have demonstrated that HFD consumption decreases plasma oesteocalcin concentrations and bone mass, and it increases plasma CTX-1 concentrations [50]. This indicates that there is an adverse effect of the HFD, which potentially favors bone breakdown as the CTX-1 concentrations are increased. A potential mechanism to explain these findings is the increased presence of adipocytes and pro-inflammatory cytokines causing the suppression of osteoblasts and increasing osteclastogenesis [21], which requires further investigation. However, in the present study, the plasma inflammatory cytokines could not be extrapolated as the plasma concentrations of TNF-α, IL-6, IL-1α, ILβ, and IFNγ were out of range of the assays (data not shown). It is important to note that despite these changes in response to high-fat feeding, it did not induce an obesity phenotype in our model. Therefore, it is likely that a diet with a higher fat content, closely mimicking a Western diet, is needed to further challenge the growth restricted dams to reveal the adverse effects of obesity on bone health. Despite these changes in bone markers following high-fat feeding, these changes were not reflected in our pQCT results; rather, the growth restricted females remained with bone deficits that were not further exacerbated by the high-fat feeding.

Of interest is the data from our mechanical testing, whereby sedentary HFD females had an increased peak force and bending stress, which was consistent with previous findings [51] and was contrary to a study in obese Zucker rats [52]. These differences were likely due to dietary intervention in an attempt to induce a model of obesity in the present study and in the study by Lau et al. [51], as compared to the genetic obesity model used by Mathey et al. [52]. Nevertheless, the mechanism behind the increased peak force and bending stress in the sedentary HFD females remains unknown. However, the aforementioned increase in the CTX-1 plasma concentrations in sedentary HFD dams suggests that bone turnover favors resorption. Moreover, there was no indication in the pQCT or histomorphometry data that high-fat feeding improved bone health. Nevertheless, our histomorphometry data was limited as we focused on the static measures of trabecular, rather than cortical, bone. Furthermore, fluorescent labelling for the dynamic histomorphometry of the dams was not possible as the study had separate fetal outcomes. 

### 4.3. Exercise Effects on Bone Health

Many studies have reported the effects of treadmill exercise on bone [35,37,52,53,54]; however, there is limited evidence of the effects of exercise in any model of growth restriction. Importantly, this study demonstrates, for the first time that the bone deficits in sedentary restricted females were prevented with exercise, which was independent of the changes in osteocalcin and CTX-1 concentrations; suggesting that bone formation and resorption remain coupled. This improved bone health following exercise was consistent with other researchers who have demonstrated that treadmill exercise in healthy rats increases tibial trabecular bone volume [53], the periosteal bone formation rate of long bone [54], femoral length, and tibial BMC [37]. These previous studies also indicated that treadmill exercise increased plasma osteocalcin concentrations [37,54], which likely contributed to the improvements in bone outcomes in their models. However, in the present study, we report no changes in the plasma osteocalcin concentrations between the control and restricted exercise females. These differences in osteocalcin concentrations following exercise may be due to the increased energy expenditure [55] and exercise intensity [53] and duration [53,54] in the previous studies compared to the present study. Another potential cause for these differences is that osteocalcin concentrations would have increased at an earlier time point than that at measurement, which may have occurred during the pre-pregnancy exercise period when intensity and duration were consistent. However, it is important to note that despite exercise improving the bone deficits in restricted females, the bone mechanical properties were not increased, which was in contrast to studies that reported increased maximal load and moment of inertia following tower climbing exercises [56]. The exact mechanism causing the exercise improvements in the maternal skeleton of growth restricted females has yet to be clarified; however, it is likely that pre-pregnancy exercise plays an important role in restoring bone deficits. During late pregnancy, the growth restricted females that exercised had increased bone parameters, compared to their sedentary counterparts, indicating that exercise assisted in increasing the bone content and strength. Additionally, it is unknown whether the benefits of exercise training will persist or whether the maternal skeleton will revert to the phenotype observed in the sedentary restricted females post-pregnancy. 

## 5. Conclusions

The novel aspect of this study is the improvement in the bone parameters of growth restricted females that exercised prior to and during pregnancy. These findings have the potential to be translated to human pregnancies that are at risk of adverse pregnancy adaptations, including females that were born growth restricted. Furthermore, our data demonstrates that endurance exercise is beneficial to the maternal skeleton, which can be a modifiable lifestyle factor adopted by females who experience adverse bone health.

## Figures and Tables

**Table 1:** F1 Control and restricted postnatal body weights. Postnatal body weights on days 1, 7, 14, and 35. Statistical significance (*p* < 0.05) determined by the Student’s unpaired t-test; * *p* = 0.0001 vs. control. Data is presented as mean ± SEM (sibling averages).

Postnatal Weight (g)	Control	Restricted
PN1	4.22 ± 0.04	3.49 ± 0.02 *
PN7	9.88 ± 0.16	7.99 ± 0.17 *
PN14	22.23 ± 0.26	18.83 ± 0.38 *
PN35	76.36 ± 0.62	68.36 ± 0.80 *

**Table 2:** Body weight measurements of the F1 females. *p*-values in italics indicate the statistical significance (*p* < 0.05) determined by two-way ANOVA to identify the differences between treatment (control and restricted) and diet (chow and HFD) within each exercise regime. A separate two-way ANOVA was then performed to identify the exercise and diet effects within each treatment (control and restricted). NS represents no statistical significance. Data is presented as mean ± SEM (n = 8–12/group).

				Two-Way ANOVA		
		Chow	HFD	Treatment	Diet	Interaction
**15 week Body Weight (g)**						
Sedentary	Control	232 ± 4.0	239 ± 7.0	*p* = 0.0001	NS	NS
	Restricted	213 ± 2.0	217 ± 7.8			
Exercise	Control	225 ± 4.1	242 ± 2.3	*p* = 0.020	*p* = 0.0001	NS
	Restricted	217 ± 2.6	234 ± 2.5			
**19 week Body Weight (g)**						
Sedentary	Control	248 ± 4.5	266 ± 7.0	*p* = 0.001	*p* = 0.026	NS
	Restricted	230 ± 2.4	240 ± 8.3			
Exercise	Control	258 ± 3.1	275 ± 5.3	*p* = 0.022	*p* = 0.0001	NS
	Restricted	247 ± 3.3	269 ± 2.1			
**Mating Body Weight (g)**						
Sedentary	Control	258 ± 4.7	270 ± 8.0	*p* = 0.002	NS	NS
	Restricted	233 ± 6.0	249 ± 7.5			
Exercise	Control	264 ± 3.7	280 ± 6.8	*p* = 0.042	*p* = 0.001	NS
	Restricted	251 ± 4.3	272 ± 4.5			
**E20 Body Weight (g)**						
Sedentary	Control	347 ± 5.3	360 ± 10.0	*p* = 0.0001	*p* = 0.030	NS
	Restricted	314 ± 4.1	335 ± 8.6			
Exercise	Control	346 ± 3.8	377 ± 7.7	*p* = 0.011	*p* = 0.0001	NS
	Restricted	332 ± 5.7	359 ± 5.9			
**Pregnancy Weight Gain (g)**						
Sedentary	Control	89 ± 3.5	90 ± 4.1	*p* = 0.027	NS	NS
	Restricted	76 ± 2.1	87 ± 2.3			
Exercise	Control	82 ± 1.8	97 ± 3.1	NS	*p* = 0.006	NS
	Restricted	81 ± 2.9	87 ± 5.4			

**Table 3:** Histological analysis of the right femur. There were no statistically significant findings (*p* > 0.05) determined by the two-way ANOVA to identify the differences between treatments (control and restricted) and diets (chow and HFD) within each exercise regime. A separate two-way ANOVA was then performed to identify the exercise and diet effects within each treatment (control and restricted). NS represents no statistical significance. Data is presented as mean ± SEM (n = 4/group).

				Two-Way ANOVA		
		Chow	HFD	Treatment	Diet	Interaction
**R. Femur Length (mm)**						
Sedentary	Control	34.3 ± 0.2	34.2 ± 0.2	NS	NS	NS
	Restricted	33.5 ± 0.2	33.4 ± 0.2			
Exercise	Control	34.9 ± 0.1	34.6 ± 0.3	NS	NS	NS
	Restricted	34.1 ± 0.1	34.6 ± 0.2			
**Bone Area (%)**						
Sedentary	Control	16 ± 3.24	22.7 ± 2.2	NS	NS	NS
	Restricted	18.6 ± 2.4	22 ± 2.9			
Exercise	Control	19.5 ± 1.6	21.7 ± 2.7	NS	NS	NS
	Restricted	19 ± 1.3	20.5 ± 3.7			
**Calcified Cartilage (%)**						
Sedentary	Control	9.3 ± 2.2	9.5 ± 1.1	NS	NS	NS
	Restricted	6.2 ± 2.3	8.4 ± 2.3			
Exercise	Control	6.8 ± 1.8	9.5 ± 0.04	NS	NS	NS
	Restricted	6.6 ± 1.6	10.3 ± 1.5			
**Growth Plate (μm)**						
Sedentary	Control	121 ± 5.8	123 ± 6.4	NS	NS	NS
	Restricted	138 ± 14.6	129.6 ± 6.2			
Exercise	Control	126 ± 8.1	128.8 ± 2.9	NS	NS	NS
	Restricted	116 ± 6.5	123 ± 11.5			

**Table 4:** Mechanical testing of the left femur. *p*-values in italics indicate statistical significance (*p* < 0.05) determined by two-way ANOVA to identify the differences between treatments (control and restricted) and diets (Chow and HFD) within each Exercise regime. A separate two–way ANOVA was then performed to identify the exercise and diet effects within each treatment (control and restricted). NS represents no statistical significance. Data is presented as mean ± SEM (n = 8–12/group).

				Two-Way ANOVA		
		Chow	HFD	Treatment	Diet	Interaction
**L. Femur Length (mm)**						
Sedentary	Control	34.4 ± 0.2	34.0 ± 0.3	NS	NS	NS
	Restricted	33.5 ± 0.2	33.5 ± 0.2			
Exercise	Control	34.5 ± 0.2	34.7 ± 0.2	NS	NS	NS
	Restricted	34.0 ± 0.2	34.3 ± 0.2			
**Peak Force (N)**						
Sedentary	Control	263.5 ± 16.0	293.9 ± 10.9	NS	*p* = 0.003	NS
	Restricted	247.2 ± 19.5	287.8 ± 12.9			
Exercise	Control	313.2 ± 2.8	286.1 ± 18.1	NS	NS	NS
	Restricted	270.9 ± 12.3	299.0 ± 22.5			
**Bending Stress (× 10^6^ m^2^)**						
Sedentary	Control	107 ± 6.6	117 ± 4.4	NS	*p* = 0.003	NS
	Restricted	100 ± 5.7	125 ± 4.6			
Exercise	Control	115.5 ± 8.5	115.1 ± 6.9	NS	NS	NS
	Restricted	109.6 ± 4.5	122.3 ± 9.1			
